# Molecular events associated with epithelial to mesenchymal transition of nasopharyngeal carcinoma cells in the absence of Epstein-Barr virus genome

**DOI:** 10.1186/1423-0127-16-105

**Published:** 2009-11-24

**Authors:** Jung-Chung Lin, Shuen-Kuei Liao, En-Huei Lee, Man-Shan Hung, Yiyang Sayion, Hung-Chang Chen, Chen-Chen Kang, Liang-Sheng Huang, Jaw-Ming Cherng

**Affiliations:** 1 Laboratory Branch, Division of Viral Hepatitis, Centers for Disease Control and Prevention, 1600 Clifton Road NE, Atlanta, Georgia, USA; 2 Graduate Institutes of Clinical Medical Sciences and Biomedical Sciences, College of Medicine, Chang Gung University, Taoyuan, Taiwan; 3 Department of Medical Research, Mackay Memorial Hospital, Taipei, Taiwan; 4 AsiaGen, Southern Taiwan Science Park, Tainan, Taiwan; 5 Department of Medical Biotechnology and Laboratory Science, College of Medicine, Chang Gung University, Taoyuan, Taiwan; 6 Department of Internal Medicine, Chung Shan Medical University Hospital/Chung Shan Medical University, 110, Jianguo N Road, Sec 1, Taichung, Taiwan

## Abstract

Epithelial-mesenchymal transition (EMT) is an important process in tumor metastasis. The EMT-related events associated with metastasis of NPC in the absence of EBV have not been elucidated. We established an EBV-negative NPC cell line from a bone marrow biopsy of an NPC patient. Using a Matrigel system we isolated an invasive and non-invasive sublines, designated NPC-BM29 and NPC-BM00. NPC-BM29 acquired an invasive-like phenotype characterized by EMT, marked by down-regulation of E-cadherin and β-catenin with concomitant increased expression of Ets1. NPC-BM29 cells expressed ≥ 10-fold higher of MMP-9 than NPC-BM00 cells. NPC-BM29 cells grew better in 2% serum than NPC-BM00 cells, with a population doubling-time of 26.8 h and 30.7 h, respectively. A marked reduction in colony-formation ability of NPC-BM00 cells compared to NPC-BM29 was observed. Wound-healing assay revealed that NPC-BM29 cells displayed higher motility than NPC-BM00 and the motility was further enhanced by cell treatment with TPA, a PKC activator. Cell surface markers and tumor-associated molecules, AE3, MAK6 and sialyl-Tn, were up-regulated in NPC-BM29 cells, whereas the expression of HLA-DR and CD54 was significantly increased in NPC-BM00 cells. NPC-BM29 consistently released higher levels of IL-8 and IL-10 than NPC-BM00, with low levels of IL-1α expression in both cell lines. Higher level of VEGF production was detected in NPC-BM00 than NPC-BM29 cells. These data show that EBV is not required for exhibiting multiple metastatic phenotypes associated with EMT. More studies that target right molecules/signalings associated with the EMT may offer new therapeutic intervention options for NPC invasion and metastasis.

## Introduction

Nasopharyngeal carcinoma (NPC) is an Epstein-Barr virus (EBV)-associated malignant tumor. NPC is latently infected with the virus, and monoclonal EBV episomes are present and expressed in the tumor cells in both endemic and sporadic forms of NPC, regardless of geographic origin [1,2]. A prominent clinical characteristic of NPC is frequent involvement of cervical lymph nodes and distant organs compared with other head and neck carcinomas [3-6]. Among head-and-neck cancers, NPC is distinguished by its highly metastatic character and poor prognosis [6].

Epithelial to mesenchymal transition (EMT) is a process that was first observed in embryonic development [7] and has more recently been implicated as an underlying event in neoplastic progression [8,9]. EMT is characterized by the loss of epithelial markers and gain of mesenchymal markers, often identified in cell lines established from tumors representing different stage and grade [10,11]. Metastatic spread of cancer cells is a result of a complex cascade of cellular/molecular events. The cascade is composed of multiple sequential steps such as down-regulation of intercellular adhesion, degradation of extracellular matrix (ECM) and up-regulation of cell motility. In addition, tumor size and likelihood of metastasis are thought to depend on increased vascularity in tumors [12].

Tumor metastasis is a complex phenomenon that is the culmination of effects of numerous cellular factors. Recent studies have shown that latent membrane protein 1 (LMP-1) of EBV induces the expression of a series of cell-invasiveness and angiogenic factors, such as matrix metalloproteinase 9 (MMP9) [13], MMP1 and MMP3 [14], c-Met and ets-1 [15], fibroblast growth factor 2 [16], vascular endothelial factor [17], hypoxia-inducible factor 1a [18], MUC1 [19], Siah 1 [20], Twist [21] and ezrin [22]. However, unlike *in vivo *growth, EBV genome is commonly lost during the establishment of NPC cell lines from biopsies or xenografts [23], implying that EBV is not necessary for maintaining the growth of carcinoma cells *in vitro*. Lack of suitable EBV-negative NPC cell lines with metastatic potential leads to poor understanding of the molecular events associated with NPC metastasis.

In this study we first attempted to separate the invasive and non-invasive populations from an EBV-negative NPC tumor cell line derived from a bone marrow lesion [24]. Secondly, we characterized the EMT morphologic changes and identified the underlying biomarkers linked to EMT that were associated with the invasiveness and metastasis.

## Materials and methods

### Cell lines and culture

NPC-BM1 is an epithelial cell line established from a bone marrow biopsy of a female Taiwanese patient with NPC [24]. The cells were originally propagated in RPMI-1640 medium but were adapted to growth in Dulbecco's modified Eagle medium (DMEM) supplemented with 2 mM L-glutamine, 0.1 mM non-essential amino acids plus 100 IU/ml penicillin, 100 μg/ml streptomycin, 0.25 μg/ml amphotericin (GIBCO, CA, USA) and 10% heat inactivated fetal bovine serum (FBS).

### Matrigel invasion assay

To separate the invasive from non-invasive cells, the BioCoat Matrigel Invasion Chambers (Becton Dickinson, CA, USA) was used according to the manufacturer's protocol. Briefly, NPC-BM1 cells (1 × 10^5 ^per well) were seeded onto the filters which were coated with the reconstituted Matrigel layered at the upper compartment of each chamber and incubated with DMEM medium. The lower chamber was filled with DMEM medium supplemented with 2% FBS. The chambers were then incubated for 48 h at 37°C. After incubation, cells on the upper side of the filters were considered as being populated by non-metastasizing cells. Cells in the lower surface of the filter were considered to have invaded through the Matrigel and thereby had metastasized.

To determine the invasive potential, cells were seeded on Matrigel and control chambers. The numbers of cells on the lower surfaces were stained with Diff-Quick solution (Sigma Chemical Company, MO, USA) and counted. The percent invasion is determined by:

### Detection of EBV genome

To detect EBV DNA in NPC-BM1, NPC-BM00 and NPC-BM29, we used primers specific for the *Bam*HI W region and *EBNA*-3C by polymerase chain reaction as described previously [25,26]. The genomic DNA isolated from NPC-BM1, NPC-BM00 and NPC-BM29 cells were analyzed in parallel with B95-8 (EBV type A strain) and AG876 (type B strain) cells. Primers flanking the β-*globin *gene were used as an internal control.

### Anchorage-independent growth assay

Anchorage-independent cell growth was examined by using a soft agar assay. The assay was done in 24-well plates with a base layer containing 1.0% agar in DMEM. Then, different cell numbers (1250, 2500, 5000 and 10000) in 0.6% agar in DMEM containing different concentrations of FBS ranging from 1.25 to 10% were embedded as a second layer. The plates were incubated at 37°C for 6 days and the number of colonies after staining was counted. Data are presented as the means ± standard deviation from experiments that were performed in triplicate wells for each time point.

### Growth Rate Determination

To determine the growth rate in high (10%) and low (2%) serum conditions, cells were seeded onto a 6-well plate (5 × 10^4 ^cells per well). The cells were trypsinized and viable cells were counted daily for 8 d with the trypan blue assay in triplicate. Growth curves were plotted. The doubling times were calculated at the exponential phase of growth cycle.

### Scrape-wound migration assay

To determine the cell motility, confluent monolayers were scraped by using a plastic pipette tip. Cells that had migrated into the scraped area were observed 7 h later. To determine the effects of tumor promoting agent TPA (12-*O*-tetradecanyol-phorbol-13-acetate) on cell migration, cells were seeded onto 6-well plates at 5 × 10^5 ^cells per well in DMEM with 10% FBS. The confluent monolayers were treated with 30 ng/ml of TPA [27,28] for 24 h followed by scrape-wounding the cells which were then studied 7 h after scraping.

### Gelatin Zymography

Matrix metalloproteinase-2 (MMP-2) and MMP-9 were assayed for gelatinolytic activity by means of gelatin zymography as reported previously [13]. Briefly, cells were cultured in DMEM containing10% FBS for 3 d. The conditioned media were harvested. Following low-speed centrifugation, the supernatants were concentrated by precipitation with 70% saturated ammonium sulfate. The precipitates were harvested by centrifugation and then were dissolved in phosphate-buffered saline (PBS) and mixed with Lammli's sample buffer (50 mM Tris-HCl, pH 6.8, 10% glycerol, 1% SDS, and 0.01% bromophenol blue) in the absence of a reducing agent; these steps were taken to denature MMPs and to dissociate any complexes with tissue inhibitors of metalloproteinases. The mixture was then incubated at 37°C for 20 min. and SDS-polyacrylamide gel electrophoresis (containing gelatin at a final concentration of 0.1%) was performed. After electrophoresis, the gel was rinsed in 2.5% Triton X-100 for 1 h and then incubated for 24 h at 37°C in a solution containing 50 mM Tris-HCl, pH 7.6, 150 mM NaCl, 10 mM CaCl_2 _and 0.02% NaN_3_. The MMPs were identified following staining of the gel in 0.1% Coomassie blue R250 dissolved in 40% methanol-10% acetic acid and destaining in the same solution without Coomassie blue. Gelatinolytic activity was visualized as a clear band against a dark background of stained gelatin.

### Western blotting

This was performed to detect proteins involved in EMT (E-cadherin, β-catenin, and Ets1). Cells were lysed with a cell lysis buffer containing 10 mM Tris-HCl (pH 7.4), 100 mM NaCl, 1 mM EDTA, 0.5% deoxycholate, 1% Triton X-100, and 1 mM phenylmethylsulfonyl fluoride. The quantity of protein in the cell lysate after centrifugation was measured with a protein assay kit (Bio-Rad). The proteins were resolved by electrophoresis with a 10% SDS-polyacrylamide gel and transferred onto a PVDF membrane (Perkin-Elmer, CT, USA). The membrane was blocked with 5% non-fat dry milk in Tris-buffered saline, pH 7.4 containing 0.1% Tween-20 before incubation with antibodies overnight at 4°C, after which the blots were washed with PBS containing 0.05% Tween-20 (PBST) and further incubated with horseradish peroxidase-conjugated secondary antibodies (1:3000 dilutions) for 1 h at room temperature. Specific blot signals were visualized on an X-ray film by incubating with ECL-Plus chemiluminescence kit (Perkin-Elmer, CT, USA). The primary antibodies against E-cadherin, β-catenin, and Ets1 were purchased from Santa Cruz Biotechnology, Inc, CA, USA, and used at a dilution of 1:2000 for E-cadherin and β-catenin, and 1:100 for Ets-1.

### Immunophenotyping

Various cellular components were examined for surface and cytoplasmic expression by immunofluorescence and flow cytometry using a panel of specific monoclonal antibodies (mAbs) including anti-HLA-A, B, C (clone W6/32; BD-PharMingen; San Diego, CA), HLA-DR (DK22, DAKO, Glostrup, Denmark), EMA (E29, DAKO), AE1 (462-01, Signet, Dedham, MA, USA), AE3 (464-01, Signet), MAK6 (28-001, Zymed, San Francisco, CA, USA), CK7 (OV-TL12/30, DAKO), CK20 (KS20.8, DAKO), EpCAM (323/A3, Thermo Scientific, Fermont, CA, USA), E-cadherin (67A4, Biodesign, Saco, ME, USA), Sialyl-Tn (49H.8; Dr. BM Longrenecker, Biomira, Edmonton, CA, USA), Lewis Y (Le^y^; ABL364; Dr H Loibner, Sandoz, Vienna, Austria); BH8.23 (Epthelial cell-associated antigen, M_r_: 50-55 kDa,. S-K Liao, unpublished), CEA (COL-1, NEOMAKERS, Fremont, CA, USA), vimentin (V9; DAKO); VEGF (G153-694; BD-PharMingen), CD44s (SFP22; BenderMed System, Vienna, Austria), CD54 (6.5B5; DAKO); and CD58 (AICD58, Beckman Coulter, Brea, CA, USA). The final working concentrations of these reagents used in immunofluorescence and flow-cytometric analysis were 5 μg/ml or diluted to a concentration according to the manufacturer's instructions.

Cells harvested from cultures were washed with PBS containing 2% FBS. For cytoplasmic expression, cells were first fixed by 1% paraformaldehyde at 4°C for 20 min, followed by permeablization with pre-cold acetone for 3 min. Cells were incubated with specific primary mAb for 30 min at 4°C. After washing, cells were then incubated with fluorescein-isothioeyanate (FITC)-conjugated goat anti-mouse IgG polyclonal antibodies (DAKO) for 30 min at 4°C. For surface antigen expression, viable singly dispersed cells were directly incubated with the primary mAb. Following a brief washing, cells were incubated with FITC-conjugated goat polyclonal Ab as for cytoplasmic antigen staining. After washing off the excess Ab, cells for both surface and cytoplasmic antigens were fixed with 1% paraformaldehyde and then analyzed on a FACscan flow cytometry machine (Becton Dickinson Co., CA, USA) to determine the level of immunofluorescent signal quantitatively. The results were expressed as % positive cells and relative mean fluorescence intensity (MFI). Proper controls were used such as replacing the second antibodies with PBS, and replacing the primary mAb with purified normal mouse IgG (NMIgG) or an isotype-matched monoclonal mouse IgG.

### Immunohistochemistry

Sections (5-μm in thickness) of OCT embedded tumor blocks obtained from xenografts were placed on gelatin-coated slides, air dried, and fixed in chilled acetone (4-5°C). Slides were washed once in PBS and stained with mAbs using tha Avidin-biotin-peroxidase complex (ABC) methods (Vectastain ABC kit, Vector Laboratories, Burlington, CA) according to mauufacturer's instructions. Results were scored as described previously (32). To perform immunocytochemistry on acetone-fixed cytospin cells of monodispersed bone marrow cells from the NPC patients, similar immunostaining procedure for xenograft tissues was used.

### Cytokine array

To compare the expression levels of cytokines associated with tumor cell growth, the following cytokines in the Q-Plex™ Human Cytokine Array (Quansys Biosciences, San Diego, CA, USA) were measured: IL-1α, IL-1β, IL-2, IL-4, IL-5, IL-6, IL-8, IL-10, IL-13, IFN-γ, TNF-α, and TNF-β. The kit provides 12 distinct capture antibodies absorbed to each well of a 96-well plate in a defined array. Once the ELISA protocol was completed, the Q-Plex™ Array was imaged using a CCD imaging system (Vilber Lourmat, France) to capture the chemiluminescent signal. Similarly, VEGF was separately analyzed by a VEGF ELISA kit (R&D Systems, Minneapolis, MN). The assay was applied to media from cells that had been cultured with serum-free DMEM sampled on d 1, 3, and 5.

### Statistical analysis

The data represent the mean ± standard deviation from 3 independent experiments. Statistical analyses were performed by using 2-tailed Student *t *tests at a significance level of *p *< .05

## Results

### Isolation of invasive cell populations

Although NPC-BM1 originated from the bone marrow, the primary site was in the nasopharyngeal cavity. To assess the metastatic potential of this tumor cell, we attempted to separate the cells possessed invasive characteristics from those that did not possess such characteristics. We used the Matrigel Invasion Assay System which provides cells with the conditions that allow assessment of their invasive property *in vitro*. Under these assay conditions, we have isolated a subline, NPC-BM18, from cells that had migrated to the lower surface of the filter. The cells remained on the upper surface of the membrane was considered as non-invasive cells and were designated NPC-BM00. The NPC-BM18 subline was expanded and further subjected to one more round of selection through Matrigel; the resulting cells were designated NPC-BM29.

**Figure 1:**
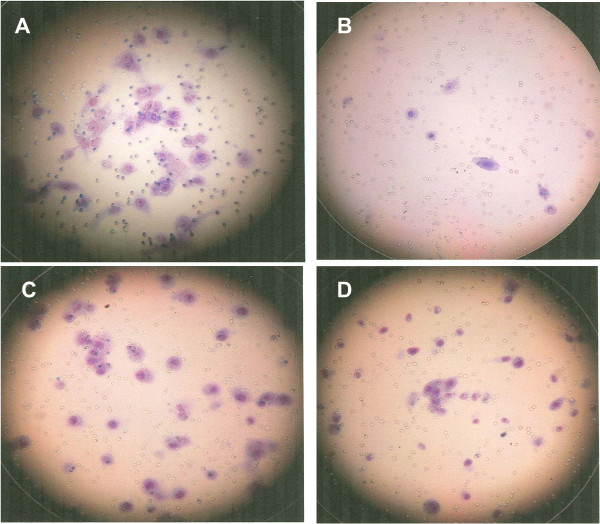
**Selection of invasive cells by Matrigel Invasion Chambers**. NPC-BM1 cells were seeded onto the filters which were coated with the reconstituted Matrigel of the upper compartment of each chamber and incubated with DMEM. After incubation, cells on the upper side of the filters were removed and cells in the lower surface of the filter were stained with Diff-Quick solution for counting. A, invasive cells (NPC-BM18) after the first round of selection; B, non-invasive cells (NPC-BM00); C, invasive cells (NPC-BM29) after the second round of selection; D, the parental cells (NPC-BM1).

**Figure 2:**
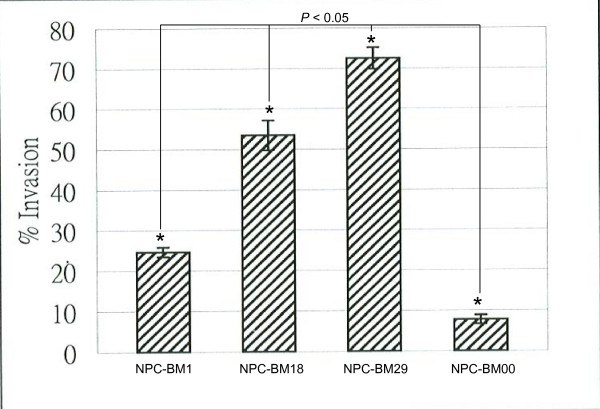
**Quantitation of migrated cells**. Cells (1 × 10^4^) were plated on the Matrigel. After 48 h, the invasive cells were stained with Diff-Quick solution. The number of invasive cells was counted from three different fields.

NPC-BM29 (Fig. 1 panel A) exhibited higher invasive capability than that of NPC-BM18 (panel C), while the parental cells NPC-BM1 (panel D) showed moderate capability of invasion. However, only limited numbers of NPC-BM00 cells (panel B) were able to migrate to the lower surface of the membrane. A quantitative analysis of the invasion assay is shown in Fig. 2.

### Detection of EBV DNA by PCR

**Figure 3:**
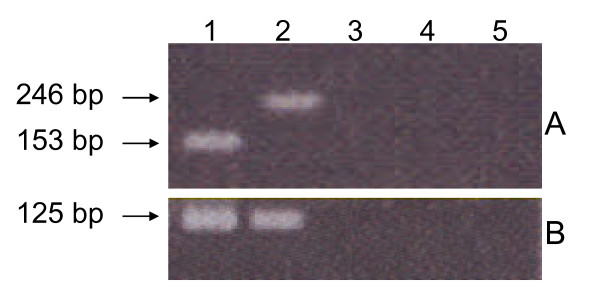
**Detection of EBV DNA by polymerase chain reaction**. Panel A, a fragment of 153 bp and 246 bp of EBNA-3C were detected in B95-8 (lane 1) and AG876 (lane 2); NPC-BM1 (lane 3), NPC-BM00 (lane 4) and NPC-BM29 (lane 5) were all negative for EBNA-3C. Panel B, an expected fragment of 125 bp of *Bam*HI W were detected in B95-8 (lane 1) and AG876 (lane 2), but not in NPC-BM1, NPC-BM00 and NPC-BM29.

PCR results for the detection of EBV DNA in NPC-BM1, NPC-BM00 and NPC-BM29 are shown in Fig. 3. The primers that were chosen to amplify the *EBNA*-3C gene encompass a strain-specific deletion [25]. Amplification with EBNA-3C primers resulted in the expected 153 bp fragment from B95-8 (Fig. 3A, lane 1) and a 246 bp fragment from AG876 (Fig. 3A, lane 2). In contrast, the EBV DNA was not detected in the parental cell line NPC-BM1 (lane 3) neither in the sublines, NPC-BM00 (lane 4) and NPC-BM29 (lane 5). The *Bam*HI W region is repeated 7 - 12 times in the EBV genome, making it a sensitive target for detecting EBV in samples where small viral copy numbers might be expected. The expected size fragment (125 bp) of *Bam*HI W with the primers used was detected only in the control samples (Fig. 3B, lanes 1 and 2), but were not detected in NPC-BM1, NPC-BM00 and NPC-BM29 (lanes 3, 4, and 5).

### Morphological changes

**Figure 4:**
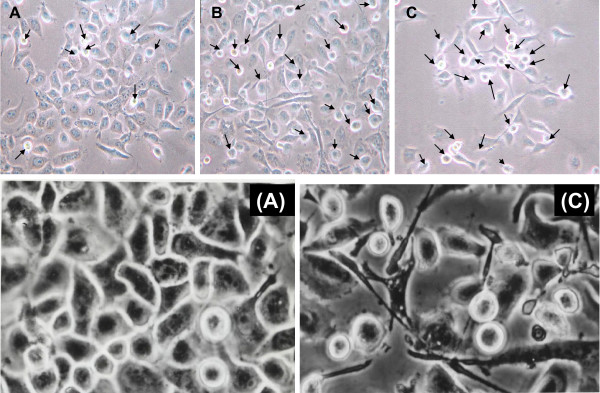
**Morphological changes of NPC-BM1 cells after separation of invasive from non-invasive cells**. Cells were plated in low density and photographed after 36 h using light microscopy (× 200 magnification). Panel A, parental cells (NPC-BM1); B, cells (NPC-BM18) after the first round of selection; C, cells (NPC-BM29) after the second round of selection. NPC BM1 exhibited typical well-attached polygonal epithelial cell morphology with few round and packed appearance cells (arrows) representing EMT morphology. These EMT cells increased in NPC-BM18 and NPC-BM29. The epithelial to mesenchymal transition (EMT) is characterized by the loss of epithelial characteristics and the gain of mesenchymal attributes in epithelial cells. Panels (A) and (C) are the higher power view of panels A and C. Magnification 450 ×.

It has been reported that "epithelial to mesenchymal transition" (EMT), the process of transdifferentiation from epithelial type to mesenchymal phenotype, is one of the major events during the acquisition of the invasive phenotype in tumors of epithelial origin. We studied whether the more invasive cells also acquired the EMT morphology. Under the light microscopy, we found that the morphology of NPC-BM1 (Fig. 4A) exhibited a typical, well-attached polygonal epithelial cell morphology with few round and packed appearance cells (arrows) representing EMT morphology. In contrast, NPC-BM18 (Fig. 4B) was heterogeneous, ranging from refractile, spindle-shaped cells with monopolar and occasional bipolar, cytoplasmic processes, to elongated fibroblast-like cells, with increasing numbers of EMT cells (arrows). Upon the second round of selection by Matrigel, the numbers of EMT cells were much enriched in NPC-BM29 (Fig. 4C).

### Cell proliferation in low serum medium and soft agar assay

**Figure 5:**
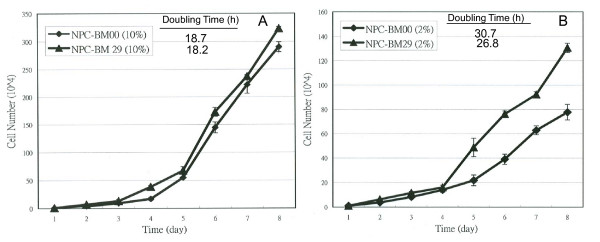
**Effect of low-serum medium on cell proliferation of NPC-BM00 and NPC-BM29 cells**. To compare the growth rate, both NPC-BM00 and NPC-BM29 were seeded and cultured in 2% FBS. The cell proliferation was monitored daily for 8 days. NPC-BM29 grew better in low serum medium than NPC-BM00.

NPC-BM00 and NPC-BM29 were routinely maintained in the DMEM supplemented with 10% FBS. Under these culture conditions, both NPC-BM00 and NPC-BM29 grew at the similar rate (Fig. 5A). The population doubling time for NPC-BM00 and NPC-BM29 were approximately 18.7 h and 18.2 h, respectively, with no statistically significant difference (*p *> .05). Upon transferring the cells into a low serum (2% FBS) growth medium, the NPC-BM29 cells exhibited an increase in growth rate compared to NPC-BM00 cells (Fig. 5B). The population doubling time was approximately 26.8 h for NPC-BM29 cells and 30.7 h for NPC-BM00 cells (*p *< .05). The total cell numbers at the saturation density in 10% serum compared to that of in 2% serum were approximately 2.5- and 3.7-fold for NPC-BM29 and NPC-BM00, respectively.

**Figure 6:**
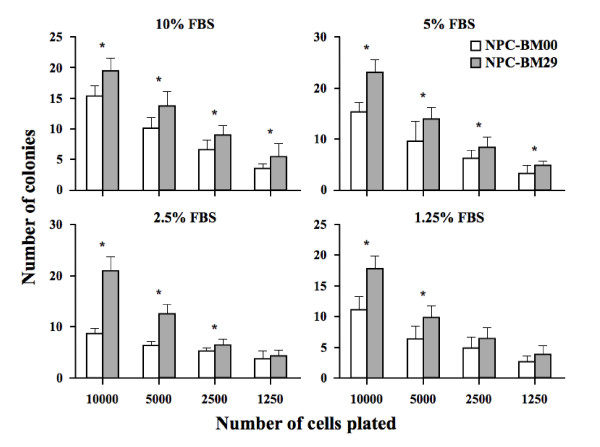
**Anchorage-independent cell growth by soft agar assay**. Soft agar assay was used to compare the colony forming ability of NPC-BM00 and NPC-BM29 cells in medium containing different concentrations of fetal calf serum (FBS) and cell numbers. Both the NPC-BM00 and NPC-BM29 cells were seeded with 1250, 2500, 5000 and 10000 cells per well in 24-well plates. The colonies were counted after 6 days of culture. Data are presented as the means ± standard deviation from experiments that were performed in triplicate wells. * referred to the level of significance at *P *< .05 and # *P *> .05.

Anchorage-independent growth assays revealed a marked reduction in colony-formation ability of NPC-BM00 cells compared to NPC-BM29 cells (Fig. 6). The most significant difference in colony forming ability was observed in the wells plated with 5000 and 10000 cells grown in 2.5% FBS.

### Determination of cell motility and TPA effect

**Figure 7:**
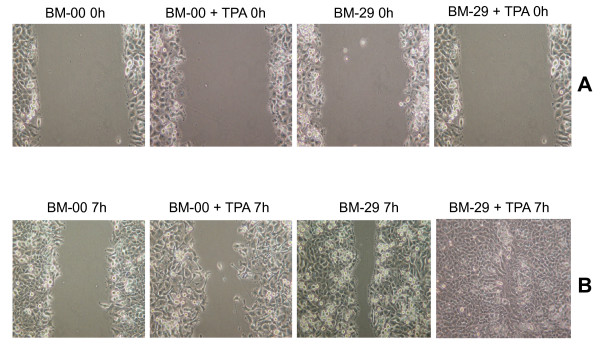
**Scrape-wound migration assay**. Confluent monolayers of NPC-BM00 and NPC-BM29 were grown in the presence and absence of tumor promoting agent TPA and were scraped by a plastic pipette. The wound-induced cell motility was observed after 7 h. Panel A, NPC-BM00 and NPC-BM29 cells in the presence and absence of TPA at 0 h after scraping; Panel B, cell motility 7 h after scraping. Magnification, 150 ×.

To compare the motility of NPC-BM00 and NPC-BM29, cells were cultured in the presence and absence of TPA and the scrape-wound assay was performed next day. Cells migrating into a wounded area were studied 7 h after scraping. As shown in Fig. 7A, the sham-treated control of NPC-BM00 cells showed slower migration into the wounded area than that of NPC-BM29 cells (Fig. 7B). In the presence of TPA, both NPC-BM00 and NPC-BM29 showed increased rate of cell migration into the wounded area. However, NPC-BM29 consistently exhibited much faster motility than that of NPC-BM00. Cells at leading edge did not adhere to each other tightly as seen in the sham-treated controls in both cases.

### Expression of metalloproteinases and invasion

**Figure 8:**
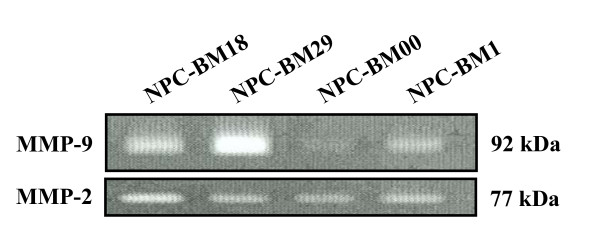
**Differential expression of MMP-9 by the NPC-BM1 parental line and its sublines (NPC-BM00. --BM18, -BM280) detected by gelatin zymography**. Cells were cultured for 3 days and the condition media were harvested and processed for gelatin zymography analysis as described in Materials and Methods. Lane 1, conditioned medium from NPC-BM18; lane 2, from NPC-BM29; lane 3, from NPC-BM00; lane 4, from parental cell NPC-BM1.

As type IV collagen is one of the integral components of basement membrane (BM), the uncontrolled expression of two type IV collagenases, MMP-2 and MMP-9, is believed to play a critical role in the invasion of BM by tumor cells [29]. The release of MMP-2, MMP-9, or both has been associated with metastasis in a variety of tumors. To correlate the epithelial-mesenchymal transition changes with the expression of MMP-2 and MMP-9, we harvested the conditioned medium for gelatin zymography analysis. Fig. 8 shows that NPC-BM1 cells secreted a small amount of MMP-9 into the culture medium (lane 4). In contrast, the amount of MMP-9 released into the culture medium was increased in NPC-BM18 cells (lane 1) and further increased in NPC-BM29 (lane 2) after a second round of selection. However, trace amounts of MMP-9 were detectable from the medium released from the non-invasive cells (NPC-BM00) (lane 3). The amount of MMP-2 secretion among these cell clones was not significantly different.

### Downregulation of E-cadherin and β-catenin

**Figure 9:**
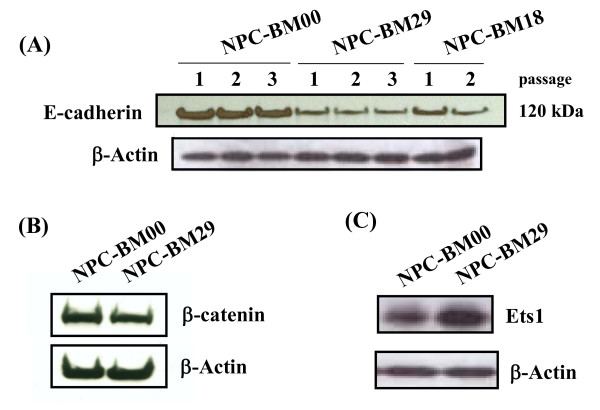
**Down-regulation of intracellular adhesion molecules E-cadherin and -catenin in NPC-BM clones**. E-cadherin and β-catenin in cell lysates of NPC-BM00, NPC-BM29 and NPC-BM18 were analyzed by Western blot using monoclonal antibodies specific for these molecules. Specific Western blot signals were visualized on an X-ray film by incubating with ECL-Plus chemiluminescence reagents. Panel A, E-cadherin; panel B, -catenin. The expression of E-cadherin remained unchanged in cells of different passages. Upregulation of transcriptional factor Ets1 was detected in NPC-BM29 cells (panel C). Both NPC-BM00 and NPC-BM29 cells were processed for Western blot analysis the same way as done for E-cadherin and -catenin except the monoclonal antibody specific for Ets1 was used.

We studied whether the expression of E-cadherin and its associated protein, β-catenin, correlated with the morphological changes and invasive properties of NPC-BM00 and NPC-BM29 cells. In Western blot analysis we found that the expression of E-cadherin was decreased in NPC-BM29 cells as compared to that expressed in the non-invasive NPC-BM00 cells (Fig. 9A.). It should be noted that the expression of E-cadherin remains unchanged in different passages within the same cell line. Concomitant with the downregulation of expression of E-cadherin, the expression of β-catenin was also decreased in NPC-BM29 (Fig. 9B).

### Overexpression of Ets1 in NPC-BM29 cells

Ets1 is a member of Ets transcription factor family and recognizes specific nucleotides sequences with a GGAA/T core sequence [30]. Expression of Ets1 in tumor cells has been found to correlate with the grade of invasiveness [31]. To compare the expression level of Ets1, we performed Western blot analysis using Ets1 monoclonal antibody. As shown in Fig. 9C, the expression of Ets1 in NPC-BM29 was markedly increased.

### Contrasting immunophenotypic characteristics between NPC-BM00 and NPC-BM29

A panel of monoclonal antibodies specific for cell surface markers and cellular tumor-associated molecules were used to characterize NPC-BM00 and NPC-BM29 cells. The results of this assay are shown in Table 1. In this assay, data were collected and expressed as the % positive cells and relative mean fluorescence intensity. Among these molecules investigated, AE3, MAK6 (a mixture of cytokeratins 8, 14, 15, 16, 18, and 19), and sialyl-Tn were significantly upregulated in NPC-BM29 cells, whereas the expression of CD54 were significantly increased in NPC-BM00 cells. Interestingly, HLA-DR was selectively expressed by NPC-BM00.

**Table 1:** Immunophenotypic characteristics of two NPC variants BM-00 and BM-29 isolated from the NPC-BM1 cell line.

Monoclonal antibody	BM-00		BM-29	
	
	Cell surface	Cytoplasmic	Cell surface	Cytoplasmic
HLA-A, B, C	96.6 (70.0)	99.5 (98.8)	98.2 (52.9)	93.2 (40.1)
HLA-DR	37.9 (43.7)	44.2 (38.9)	0.16 (42.3)	0.8
NMIgG	1.0	1.6	1.0	0.4
PBS	0.9	0.6	0.9	1.0
EMA	8.0	82.7 (24.5)	5.9	75.2 (18.9)
AE1	ND	54.5 (36.5)	ND	40.5 (23.2)
AE3	ND	28.5 (9.7)	ND	44.2 (8.4)
MAK6	ND	17.8 (9.7)	ND	28.7 (8.5)
CK7	ND	97.6 (57.8)	ND	99.9 (55.1)
CK20	ND	37.7 (13.7)	ND	31.8 (11.4)
EpCAM (ESA)	98.7 (54.5)	ND	93.8 (39.3)	ND
Sialyl-Tn	2.6	5.2	7.8	34.1 (14.5)
Le^y^	0.5	1.2	0.8	0.8
BH8.23(50-55 kDa)	6.5	77.3 (32.5)	6.4	95.4 (33.8)
CEA	ND	9.5	ND	4.8
Vimentin	ND	97.7 (84.6)	ND	99.9 (92.1)
VEGF	ND	98.7 (29.4)	ND	99.7 (31.8)
CD44s	89.7 (20.3)	ND	90.0 (26.8)	ND
CD54	95.7 (36.3)	ND	87.6 (23.8)	ND
CD58	80.8 (10.6)	ND	99.2 (31.0)	ND

Results are obtained by immunofluorescence and flow cytometric analysis and are expressed as % positive cells and relative mean fluorescence intensity (in the bracket). Note that those results with <10% positive cells are considered negative and thus values of mean fluorescence intensity are not given.

Abbreviations: ND, not determined; NMIgG, normal mouse Ig; PBS, phosphate-buffered saline; EMA, epithelial membrane antigen; CK, cytokeratin, EpCAM, epithelial cellular adhesion molecule; ESA, epithelial specific antigen; Le^y^. Lewis Y; CEA, embryonic carcinoma antigen; VEGF, vascular endothelial growth factor.

Immunophenotypic features of NPC-BM1 that distinguishes it as NPC is presented in Table 2. It is clearly shown that the SCID mouse xenograft of NPC-BM1 cells exhibited similar immunostaining features as the bone marrow aspirate from which the NPC-BM1 cell line was derived, aside from the fact that both tissues displayed poorly differentiated histopathology (not shown). Positive staining of HLA-DR was observed in a significant proportion of tumor cells in both tissues. In sharp contrast, HLA-DR was not expressed in the cell lines derived from primary NPC lesions, such as NPC039, NPC076, NPC-BR, CNE-1 and CNE-2, which have been maintained in our laboratory [32].

**Table 2:** Comparison of immunophenotypic features of NPC-BM1 cells and the original bone marrow aspirate

Monoclonal antibody	Xenograft (section)	Bone marrow aspirate (cytospin)
HLA-A, B, C	++++*	++++
HLA-DR	++ (focal)	++ (scattering)
NMIgG	-	-
PBS	-	-
EMA	+++	+++
AE1	++	++
CK7	++++	++++
EpCAM (EPS)	++++	++++
Le^y^	-	-

Immunostaining of the SCID mouse xenograft resulting from *s*. *c*. injection of NPC-BM1 cells and the original bone marrow biopsy (cytospin cells) from which the NPC-BM1 cell line was established.

*Results are expressed by the extent of immunostaining on tumor cells. See the footnote of Table 1 for the abbreviations of the markers tested.

### Cytokine expression profiles

**Figure 10:**
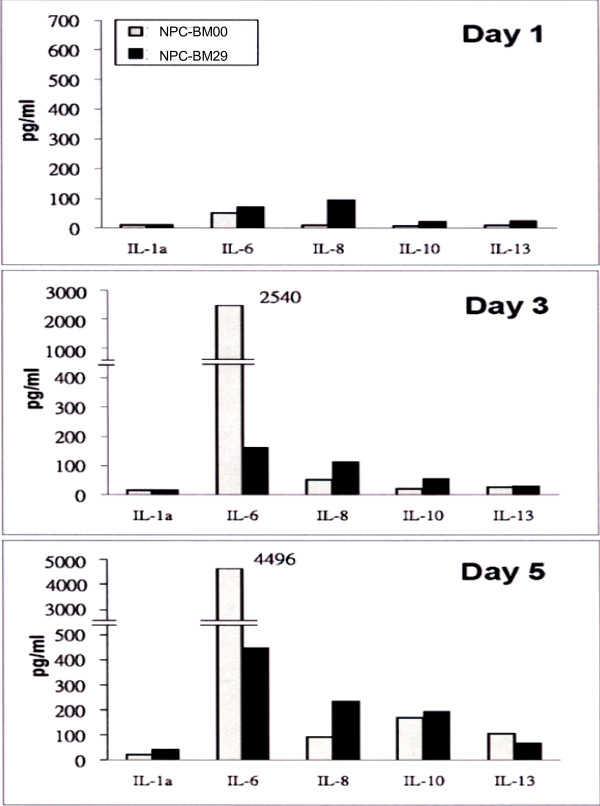
**The cytokine expression profiles of NPC-BM00 and NPC-BM29 cells**. The cultured media from NPC-BM00 and NPC-BM29 cells were collected and analyzed by the Q-Plex™ Human Cytokine Array for a panel of 12 distinct cytokines as described in the text. The cytokine expression profiles were monitored and analyzed from cultured media collected on day 1, 3, and 5.

**Figure 11:**
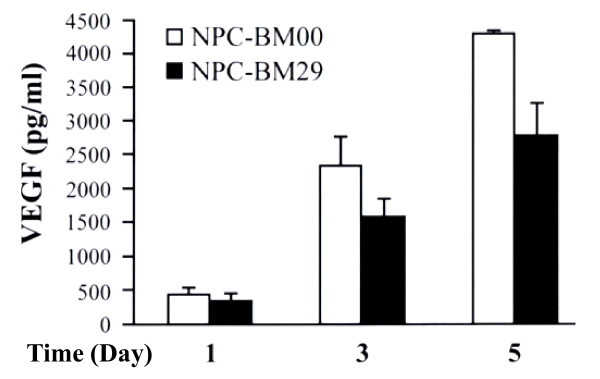
**Detection of VEGF production by ELISA**. The cultured media from NPC-BM00 and NPC-BM29 cells were collected and analyzed by ELISA using VEGF monoclonal antibody coated on the microtiter plate. The VEGF expression profiles were monitored and analyzed from cultured media collected on day 1, 3, and 5.

The cytokines released into the cultured media were measured on d 1, 3, and 5. Fig. 10 shows the cytokine expression profiles. On day 1, NPC-BM29 consistently released higher levels of IL-6, IL-8, IL-10, and IL-13 than NPC-BM00 cells. There was no difference in the expression level of IL-2a in both cell lines. On d 3 the production of IL-6 by NPC-BM00 was markedly increased to approximately 3-fold higher than that released by NPC-BM29. However, the production of IL-8, IL-10, and IL-13 on d 3 remained at similar levels as on d 1. On d 5, both cell lines maintained similar high levels of IL-6 production. Despite the fluctuation of expression level of IL-6, it is of interest to note that NPC-BM29 consistently released higher levels of IL-8 and IL-10 in a time-dependent manner throughout the entire period of assays. In contrast, NPC-BM00 cells consistently produced a higher level of VEGF than NPC-BM29 cells (Fig. 11).

## Discussion

We had previously established a cell line, NPC-BM1, derived from a bone marrow biopsy of a female Taiwanese patient with NPC [24]. Some basic differences in baseline secretion of IL-6, IL-6 receptor α and IFN-γ between NPC-BM1 and other NPC cell lines derived from primary tumors have recently been documented [33]. In the current study, this parental cell line was further separated to non-invasive and highly invasive cells, designed NPC-BM00 and NPC-BM29, respectively. After separation, the metastatic phenotype of these two cell lines was confirmed by cell morphology change suggestive of EMT. While NPC-BM00 cells exhibited typical well-attached polygonal epithelial cell morphology, NPC-BM29 was more heterogeneous and included cells that were spindle-shaped cells and those resembled fibroblasts.

EMT is characterized by the loss of epithelial characteristics and the gain of mesenchymal attributes. It has been associated with physiological and pathological processes requiring epithelial cell migration and invasion. In addition, evidence is mounting suggesting the importance of EMT pathways in the progression of carcinoma to metastasis by providing epithelial tumor cells with the ability to migrate, invade the surrounding stroma and disseminate in secondary organs [34]. It has been reported that EMT is one of the major events during the acquisition of the invasive phenotype in tumors of epithelial origin [8,35]. This process is often accompanied by expression of mesenchymal markers and loss of epithelial markers, especially E-cadherin, which is one of the most commonly observed changes in invasive and metastatic carcinomas [36,37].

In the scrape-wound assay, we showed that NPC-BM00 cells exhibit slower migration into the wounded area than its counterpart NPC-BM29. This observation was further supported by the findings demonstrating downregulation of E-cadherin and its associated protein β-catenin in NPC-BM29 cells (Fig. 9). Previously we showed that TPA is a highly potent activator of EBV genome replication [27,28]. TPA also has been shown to activate some isoforms of the important signaling enzyme, protein kinase C (PKC) [38], which interferes with both cell proliferation and cell adhesion in a variety of cell types [39]. Various studies have demonstrated the involvement of PKC in disassembly of E-cadherin-dependent cell-cell adhesion [39-42]. In the present study, we analyzed the effect of TPA on cell motility and revealed that both NPC-BM00 and NPC-BM29 showed increased rate of cell migration into the wounded area compared to the sham-treated cells but with differential rate. Activation with TPA also resulted in a decrease in the level of E-cadherin in both cases (our unpublished data). Based on these results, we conclude that PKC activation is involved in TPA-induced cell motility.

E-cadherin and β-catenin are another commonly employed index for the epithelial state. These cell adhesion molecules are localized in the adherence junctions. These cell-cell adhesion-related criteria are almost exclusively absent in mesenchymal cells. The E-cadherin plays a central part in the process of epithelial morphogenesis. Expression of this protein is downregulated during the acquisition of metastatic potential at late stages of epithelial tumor progression. In cancer, the maintenance of epithelia is lost, and dissociation of cells is associated with metastatic dissemination. Dissociation of cells can occur through a decrease in the local expression level of E-cadherin.

Invasion and metastasis are determinative features in the pathogenesis and progression of malignant neoplasms. The pathogenesis of metastasis consists of multiple, sequential, selective and interdependent steps. To establish a metastatic focus, tumor cells must detach from the primary tumor (suppression of cell-to-cell and cell-matrix adhesion), degrade and invade the extracellular matrix (ECM), increase in cell motility and enter the circulation, arrest in a capillary bed, gain entrance into organ parenchyma, proliferate and induce angiogenesis [43]. It is now well established that the processes of invasion and angiogenesis are essential for the growth and metastasis of both primary and metastatic tumors [12].

Matrix metalloproteinases (MMPs) are a family of structurally related zinc-dependent endopeptidases collectively capable of degrading essentially all components of ECM. Based on their structure and substrate specificity, MMPs are classified into subgroups of collagenases: stromelysins and stromelysin-like MMPs and other MMPs [44]. MMPs play an important role in the physiologic degradation of ECM, e.g., in tissue morphogenesis, tissue repair and in angiogenesis. MMPs also have important functions in pathologic conditions characterized by excessive degradation of ECM, such as rheumatoid arthritis, osteoarthritis, periodontitis, autoimmune blistering disorders of the skin, as well as in tumor invasion and metastasis [44-47]. MMP-1 (collagenase-1) cleaves fibrillar collagens with preference for type III collagen, which denatures into gelatin and is further degraded by other MMPs, such as gelatinases. MMP-2 (gelatinase-A) and MMP-9 (gelatinase-B) can both degrade the type IV collagen of basement membranes, the first barrier to tumor invasion. Thus, high expression levels of certain MMPs facilitate tumor cell invasion and metastasis. Our results revealed that after separation, the metastasis potential of NPC-BM29 was confirmed by increased expression of 92-kDa type IV collagenases/gelatinase B (MMP-2 and MMP-9); both enzymes are involved in tumor cell invasion and metastasis.

Ets1 is a member of Ets transcription factor family and recognizes specific nucleotides sequences with a GGAA/T core specific sequence [30]. Physiologically, Ets1 is expressed in various mesodermal derivatives, such as endothelial cells and mesenchymal cells, but is also induced in dissociating epithelial cells, during EMT process occurring in early development and oncogenic transformation to acquire invasive features [30,48]. Ets1 expression in tumor cells has been found to correlate with the grade of invasiveness in human tumor tissues [49] and correlates with a degree of invasiveness in breast cancer cell lines [48]. Ets family members were reportedly involved in the control of cell motility and invasion during normal tubular morphogenesis and cancerous scattering in mammary epithelial cells [50]. Furthermore, it has been reported that MMP9 gene promoter contains a binding site for Ets in addition to AP-1 and NF-kB, and thus Ets1 may positively regulate MMP9 gene expression [51]. Therefore, Ets1 may stimulate transcription of many genes associated with tumor invasion and metastasis, and would be an effective target in preventing invasion of malignant tumors.

Using Western blot analysis, we have identified that the expression of E-cadherin and β-catenin was decreased with the increasing metastatic potential of NPC-BM29 cells. Concomitant with the downregulation of E-cadherin and β-catenin, upregulation of the transcription factor Ets1 was detected in NPC-BM29 cells, in agreement with the central role of these molecules in the transition of EMT.

We observed that NPC-BM1 cells formed compact cell islands in tissue culture plates, remained in tight cell-cell contacts and exhibited the typical morphology of epithelial cells. In sharp contrast, NPC-BM18 and 29 cells were scattered and exhibited the typical morphology of mesenchymal fibroblasts, suggesting that the constitutive activation of β-catenin signaling might induce EMT [8].

It has been well-documented that the transformed cells do not require high concentration of serum in the culture medium to support the proliferation. In this study, we demonstrated that serum deprivation markedly retarded the growth of NPC-BM00 cells with little or no metastatic potential, but had no significant impact on NPC-BM29 cells likely to have higher metastatic potential (Fig. 5). This phenomenon suggests the possibility that NPC-BM00 cells may convert the cell phenotype toward benign, because serum-independent proliferation generally is accepted as a hallmark of tumor cells but not normal cells. Indeed, this speculation is supported further by the observation that significantly fewer colonies were formed in soft agar by NPC-BM00 than by NPC-BM29 cells (Fig. 6).

The preferential expression of sialyl-Tn by NPC-BM29 cells on one hand and the selective expression of HLA-DR by NPC-BM00 cells on the other in a significant proportion of cell population are interesting. While the implications of these results are not immediately clear, further investigations on both observations are warranted.

Angiogenesis is a key step in tumor growth, invasion and metastasis. Massive formation of blood vessels at the tumor site increases the opportunity for tumor cells to enter the circulation. Thus, microvessel density is considered to influence tumor metastasis and consequently prognosis in various human cancers [52]. VEGF, FGF basic (bFGF) and IL-8 are prominent angiogenic molecules. These molecules have been demonstrated to influence microvessel synthesis in various tumors [53,54]. The associations of neovascularization to both angiogenic and lymphatic metastases have been examined in many malignant tumors, and the contributions of angiogenic molecules such as VEGF, bFGF, and IL-8 to the metastatic potential of tumors have been well documented. The relevance of angiogenic factors to the angiogenesis of these NPC cell lines described in this study was evaluated by studying the relationships of IL-8 and VEGF expression. Our results clearly indicate that the higher level expression of IL-8 in NPC-BM29 was significantly correlated to its invasive and metastatic potential. In contrast, the upregulation of VEGF was found in NPC-BM00 cells suggesting that it may enhance the growth capability by facilitating neovascularization locally.

The roles of EBV infection in the tumorigenesis of NPC are of interest because of the close association of this virus with NPC. Recent reports indicate that EBV LMP-1 is capable of inducing a wide range of cellular factors associated with metastatic character of NPC [13-22]. However, the critical functions of EBV in NPC development remain poorly understood. It has been noted that the EBV genome is commonly lost during the establishment of NPC cell lines from biopsies or xenografts [23]. However, EBV genomes are not lost from tumors being propagated in nude mice. Such tumors retain characteristics of the original NPC including p53 negativity. The loss of EBV genomes during attempts to culture NPC *in vitro *is associated with inability to establish cell lines from original or nude-mouse passaged tumors.

It should be noted that both the parental cell line (NPC-BM1) and two sublines (NPC-BM00 and NPC-BM29) are EBV-negative (Fig. 3). This observation suggests that EBV may have other important roles *in vivo*, which may include, but are not limited to, conferring protection to the carcinoma cells from immune surveillance [55].

Overall, this study reports our observations on important cellular signaling cascades that are associated with metastasis of NPC in the absence of EBV genome. The link between morphologic changes from epithelial to mesenchymal transition and identification of the molecules associated with these changes opens up new avenues in cancer research, allowing the use of these molecular markers to pave the way for the identification of novel targets for therapeutic intervention of invasion and metastasis.

## Abbreviations

MMP: matrix metalloproteinase; TPA: 12-*O*-tetradecanyol-phorbol-13-acetate; VEGF: vascular endothelial growth factor; EMT: epithelial-mesenchymal transition; EBV: Epstein-Barr virus; LMP-1: latent membrane protein-1.

## Competing interests

The authors declare that they have no competing interests.

## Authors' contributions

Both JCL and SKL designed the study and its coordination and participated in and drafted the manuscript. EHL carried out the Western blot and cell cycle analysis. MSH performed the selection of metastasizing cells. YS carried out the gelatin zymography assays. HCC, CCK and LSH carried out the cytokine expression profiles and anchorage-independent cell growth. JMC initiated the study, performed the statistical analysis, carried out detection of EBV DNA, and coordinated during the course of the study. All authors read and approved the final manuscript.
